# Flexural Fatigue Performance of Steel Fiber Reinforced Expanded-Shales Lightweight Concrete Superposed Beams with Initial Static-Load Cracks

**DOI:** 10.3390/ma12193261

**Published:** 2019-10-06

**Authors:** Fulai Qu, Changyong Li, Chao Peng, Xinxin Ding, Xiaowu Hu, Liyun Pan

**Affiliations:** 1 School of Civil Engineering and Communications, North China University of Water Resources and Electric Power, Zhengzhou 450045, China; qfl@ncwu.edu.cn (F.Q.); dingxinxin@ncwu.edu.cn (X.D.); Xiaowu-hu@stu.ncwu.edu.cn (X.H.); 2 International Joint Research Lab for Eco-Building Materials and Engineering of Henan, North China University of Water Resources and Electric Power, Zhengzhou 450045, China; zmdpengchao@163.com; 3 Henan Provincial Collaborative Innovation Center for Water Resources High-efficient Utilization and Support Engineering, Zhengzhou 450046, China

**Keywords:** superposed beam, steel fiber reinforced expanded-shales lightweight concrete (SFRELC), flexural fatigue, stress level, crack width, flexural stiffness, fatigue life

## Abstract

Concerning the structural applications of steel fiber reinforced expanded-shales lightweight concrete (SFRELC), the present study focuses on the flexural fatigue performance of SFRELC superposed beams with initial static-load cracks. Nine SFRELC superposed beams were fabricated with the SFRELC depth varying from 50% to 70% of the whole sectional depth, and the volume fraction of steel fiber ranged from 0.8% to 1.6%. The fatigue load exerted on the beams was a constant amplitude sinusoid with a frequency of 10 Hz and a fatigue characteristic value of 0.10; the upper limit was taken as the load corresponded to the maximum crack width of 0.20 mm at the barycenter of the longitudinal rebars. The results showed that with the increase of SFRELC depth and the volume fraction of steel fiber, the fatigue life of the test beams was prolonged with three altered failure modes due to the crush of conventional concrete in the compression zone and/or the fracture of the tensile rebar; the failure pattern could be more ductile by the prevention of fatigue fracture by the longitudinal tensile rebar when the volume fraction of steel fiber was 1.6% and the reduction of crack growth and concrete strain in the compression zone; the fatigue life of test beams was sensitive to the upper-limit of the fatigue load, a short fatigue life appeared from the higher stress level and larger stress amplitude of the longitudinal rebar due to the higher upper-limit of the fatigue load. The methods for predicting the stress level, the stress amplitude of the longitudinal tensile rebar, and the degenerated flexural stiffness of SFRELC superposed beams with fatigue life are proposed. With the optimal composites of the SFRELC depth ratio and the volume fraction of steel fiber, the controllable failure of reinforced SFRELC superposed beams could be a good prospect with the trend curves of fatigue flexural stiffness.

## 1. Introduction

With the development of high-rise buildings and the increasing span and space of concrete structures, the disadvantage of conventional concrete with great self-weight becomes more acute compared to the imposed loads. This promotes the research and development of structural lightweight concrete with different kinds of lightweight aggregates categorized as natural lightweight aggregates and artificial lightweight aggregates [1,2,3,4]. Except for the savings in dead load for structure and on foundation due to the self-lightweight, structural lightweight concrete presents many advantages, including the rising of strength/weight ratio, the reduced risk of earthquake damage to a structure, superior thermal and sound insulation, and better durability [3,4,5]. However, some problems, including greater brittleness than conventional concrete for the same compressive strength, prevent the wide application of structural lightweight concrete [6,7]. One way to resolve these problems is the use of steel fibers. Based on previous research, steel fiber reinforced expanded-shales lightweight concrete (SFRELC), with market-supply sintered expanded-shales for the fine and coarse aggregates, has excellent mechanical properties, especially those related to tensile performances [8,9,10,11,12,13]. Due to lower shrinkage and reliable bond behavior with the rebar, the SFRELC can be applied to concrete structures [14,15,16,17]. To highlight the peculiarities of the tensile performance of SFRELC and the compressive property of conventional concrete, the SFRELC superposed beams and slabs were innovatively developed. The sectional characterization of this kind of superposed flexural member is composite with tensile SFRELC and compressive conventional concrete [18,19], while the fabrication is the bottom-layer of SFRELC successively followed by up-layer conventional concrete [20,21,22]. Based on the experimental studies and numerical analyses, the design methods for the flexural performances, including cracking resistance, crack width, flexural stiffness, and bearing capacity, of the reinforced SFRELC superposed beams under static loading were built up [23,24,25,26].

Civil engineering concrete structures are always subject to the actions of repeated loads. For example, industry plant structures suffer from the vibration from machines, bridges bear the vibration of rolling vehicles, and ocean structures are subject to repeated waves. This raises an important topic of research on the normal serviceability and reliability of engineering concrete structures under fatigue actions. Normally, the key points are concerned with the fatigue behaviors of structural materials, including the fatigue compressive performance of concrete and the fatigue fracture of tensile rebars [27,28,29,30,31]. At the level of structural members under fatigue load, geometric shapes, sectional composites of concrete and reinforcement, and the sectional stress distribution with different fatigue characteristics are also main factors [32,33,34,35,36,37,38,39,40]. For the reinforced SFRELC superposed beams, the shear fatigue behaviors were confirmed [34]. Results show that the maximum fatigue load controls the initial diagonal crack width and initial stress amplitude of stirrups, which has a great influence on the fatigue life; with an increase of maximum fatigue load, the diagonal crack width grows quickly and the fatigue failure of the test beams takes place with a large possibility of the fracture of stirrups; overload during fatigue is one of the main reasons for fatigue failure, which results in the sudden increase of diagonal crack width and stirrup’ stress amplitude. Except for shear fatigue performance, a study of the flexural fatigue performance is also essential for the engineering application of reinforced SFRELC superposed beams subjected to fatigue loads. As per the previous studies on the flexural fatigue behavior of steel fiber reinforced concrete (SFRC) beams [35,36,37,38,39,40], the presence of steel fibers could promote fatigue resistance to crack growth, decrease the deflection and increase the energy dissipation at failure, and prolong the fatigue life of reinforced SFRC beams. This is due to the beneficial effect of SFRC at the tensile zone in reducing the stress level of tensile rebars. Therefore, with the integrity of the horizontal interface between SFRELC and conventional concrete, good flexural fatigue behavior of the SFRELC superposed beams could be a good prospect.

Due to a lack of investigation on the flexural fatigue of SFRELC superposed beams, the experimental study was carried out in this paper. Nine beam specimens with rectangular sections were fabricated and tested by a four-point flexural test under a constant amplitude sinusoid at a frequency of 10 Hz with a fatigue characteristic value *ρ*_f_ = 0.10. The main influence factors were the SFRELC depth change as 0.5, 0.6, and 0.7 times of the whole sectional depth, and the volume fraction of the steel fiber varied as 0.8%, 1.2%, and 1.6%. The upper limit of the fatigue load was taken as the load corresponding to the maximum crack width of 0.20 mm at the barycenter of the longitudinal rebar. This crack width is the limit for reinforced concrete structure cracks within the life of normal serviceability in normal environmental conditions [41]. Based on the experimental results, the crack distribution, crack width, mid-span deflection, failure patterns, and compressive strain of conventional concrete are discussed. Methods for prediction of the stress level, stress amplitude of the longitudinal tensile rebar, and flexural stiffness degeneration with fatigue life are proposed.

## 2. Experimental Work

### 2.1. Raw Materials and Basic Properties of Concrete

Common Portland cement with a strength grade of P.O. 52.5 and class-II degree fly ash were used as binders. Their properties met the requirements of the China codes GB 175 and GB/T1596 [42,43]. The content of fly ash was 20% of the total mass of the binders. The compressive and tensile strengths of the cement were 51.7 MPa and 9.2 MPa at 28 days. The water demand of the fly ash was 95% with a fineness modulus of 3.9. Sintering expanded shale, ceramisite sand, and steel fiber were used for SFRELC. Based on the maximum density principle, sintering expanded shale was sieved in continuous grading with a maximum particle size of 20 mm. Bulk and particle densities were 827 kg/m^3^ and 1262 kg/m^3^, cylinder compressive strength was 7.4 MPa, and 1 h water absorption was 9.1%. The ceramisite sand was made from the byproduct of sintering expanded shale with a fineness modulus of 3.56 in continuous grading with a particle size of 1.6–5 mm. Bulk and particle densities were 850 kg/m^3^ and 1350 kg/m^3^, and 1 h water absorption was 9.0%. The steel fiber was of a milling type with length *l*_f_ = 36 mm, equivalent diameter *d*_f_ = 1.35 mm, and the aspect ratio *l*_f_/*d*_f_ = 26.7. Crushed limestone with continuous grading of a 5–20 mm particle size and river sand with a 2.84 fineness modulus were used for conventional concrete. Other raw materials were the high-performance water reducer with 20% water reduction and tap-water. The mix proportions of SFRELC and conventional concrete were designed initially based on the absolute volume method at a saturated surface-drying status of raw materials [44,45] and adjusted according to previous studies [16,22,27]. The water/binder ratios of SFRELC and conventional concrete were 0.384 and 0.389.

As listed in Table 1, the slump of the concrete mixture was measured by the slump cone method in accordance with China code GB50080 [46]. The workability for all fresh mixtures was good for casting. The basic mechanical properties, including the cubic compressive strength ***f*_fcu_** (***f*_cu_**), axial compressive strength ***f*_fc_** (***f*_c_**), splitting tensile strength ***f*_ft_** (***f*_t_**), and modulus of elasticity *E*_c_ of SFRELC and conventional concrete were measured by using standard cubes with a dimension of 150 mm and prisms with a dimension of 150 mm × 150 mm × 300 mm. These standard specimens were cast and cured in the same conditions accompanied by the test beams in accordance with the China codes GB50081 and GB50152 [47,48].

### 2.2. Preparation of Test Beams

**Figure 1:**
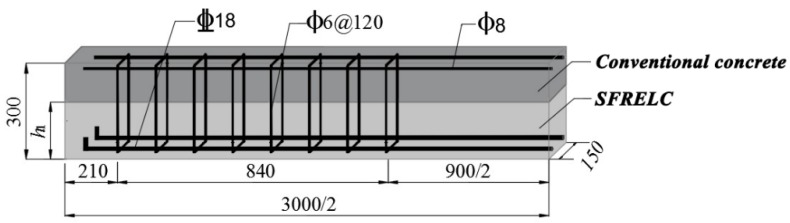
3D view of reinforced SFRELC superposed beam (unit: mm).

Nine test beams with rectangular sections were designed with the prospect of flexural failure [23,41]. The dimensions were, width *b* = 150 mm, depth *h* = 300 mm, length *L* = 3.0 m, and span *l*_0_ = 2.7 m. As presented in Figure 1, each beam had 2 Φ18 mm hot-rolled deformed HRB400 longitudinal tensile rebars, 2 Φ8 mm hot-rolled plain HPB300 longitudinal construction rebars, and Φ6 mm hot-rolled plain HPB300 stirrups with spacing of 120 mm. The yield strength and ultimate strength of the Φ18 mm rebars were 451 MPa and 564 MPa. The thickness of the concrete cover for the longitudinal tensile rebars was *c* = 25 mm. The sectional depth (*h*_1_) of SFRELC changed from 150 mm to 210 mm, corresponding to the ratio of the whole sectional depth *α*_h_ = 0.5, 0.6, and 0.7, respectively. The volume fraction of the steel fiber of SFRELC was *v*_f_ = 0.8%, 1.2%, and 1.6%. The details of the tested beams are presented in Table 2.

The procedure for the fabrication of the test beams was the same as previous studies [23,24,25,26,27]. The steel reinforcement framework was made of steel bars with designed dimensions and geometric shapes, and the formwork was assembled from formed steel plates. The mixture of concrete was mixed by a 500 L horizontal-shaft forced mixer cast into the formwork and compacted by the vibrators that hung on the outside surface of the formwork. After that, the top surface of the SFRELC was covered by a plastic film for 48 h. Then, the formwork was demolded, and the test beams were cured for 7 d by spraying water.

### 2.3. Test Method

**Figure 2:**
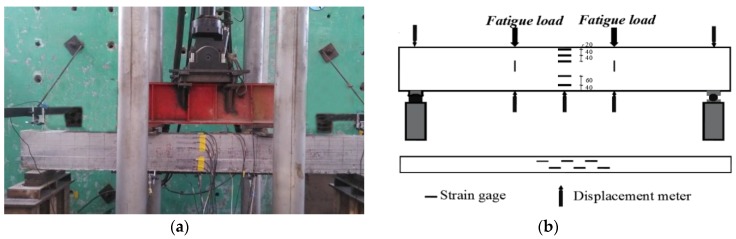
Fatigue testing devices of test beams: (**a**) live-action photo; (**b**) gauge installation.

The four-point bending tests were carried out on the test beams by an MTS fatigue testing machine. The concentrated loads were measured and controlled by the MTS loading system on the top surface of the beam, as exhibited in Figure 2. Test beams were firstly loaded under static load to make the initial cracks. To accelerate the test procedure and get failure under fatigue load, the upper limit of the fatigue load, *P*_max_, on each beam was taken as the load corresponding to the maximum crack width of 0.20 mm at the side surface barycenter of the longitudinal tensile rebars. In accordance with China code GB50152 [48], the fatigue load was taken as a constant amplitude sinusoid at a frequency of 10 Hz with the fatigue characteristic value *ρ*_f_ = 0.10. The test procedure was as follows: (1) pre-load within 20% *P*_max_ was exerted for 2–3 times to ensure the loading and data collection system worked well; (2) the static repeat load tests within *P*_max_ were conducted twice, and the fatigue test was started at the initial load of 0.1*P*_max_; (3) test data were read while the fatigue number *N* reached 5000, 10,000, 50,000, 100,000, 200,000, and 500,000; after that, data were collected at fatigue numbers with an increment of 500,000; (4) the test was conducted until the beam was damaged.

As displayed in Figure 2, the concrete strains at the mid-span section of the test beam were measured by the six strain gauges bonded on the side surface, and the strain gauges were continuously bonded on the top and bottom surfaces. The deflection of the test beam was measured by the electrical displacement meters installed on the supports and mid-span section. Crack width at the side surface barycenter of the longitudinal tensile rebars was read by using a crack visualizer with a precision of 0.02 mm. To simplify the discussion on the general variation of the compressive concrete strain, the concrete strains at the top surface of the test beams are computed as an average for each group. The width of the main cracks and the mid-span deflection of the test beams in each group are also computed as an average in this study.

## 3. Results

### 3.1. Crack Distribution and Growth

**Figure 3:**
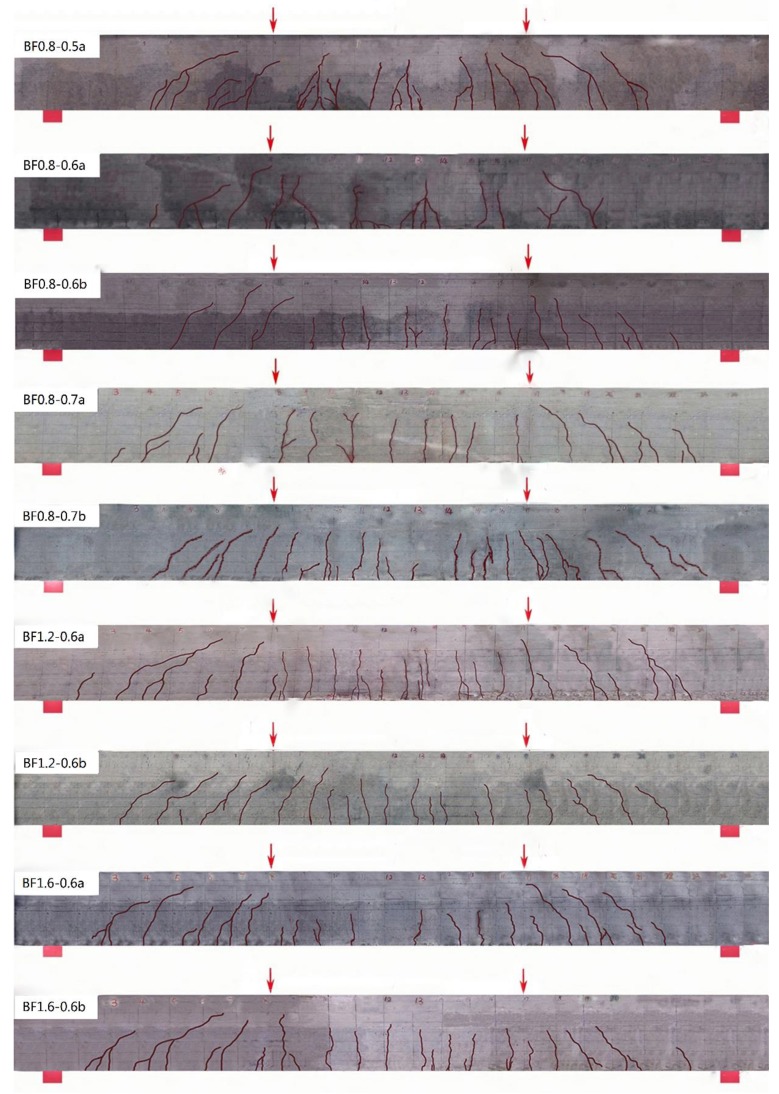
Crack distribution of test beams with different *α*_h_ or *v*_f_.

**Figure 4:**
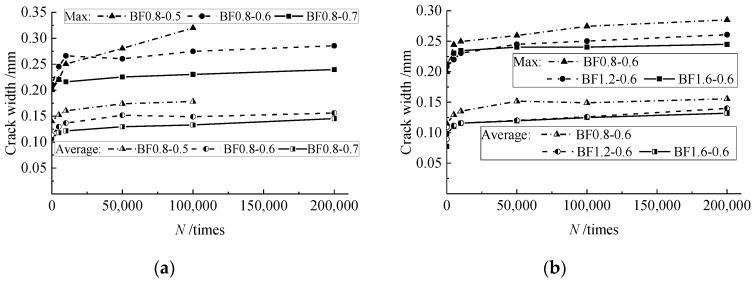
Changes of crack width affected by: (**a**) *α*_h_; and (**b**) *v*_f_.

Figure 3 presents the crack distribution of test beams before fatigue failure. The main cracks at pure bending sections almost appeared during the procedure of static loading before fatigue. Combined with the changes of the maximum and average crack width presented in Figure 4, the cracks elongated and opened rapidly under the fatigue within 5000 numbers. After that, the development of cracks became steady with some root cracks at the bottom of the test beams. 

With the increase of the SFRELC depth ratio *α*_h_, more cracks appeared on the test beams while the maximum and average main crack width decreased. In the steady development period, the maximum crack width decreased by about 11% when *α*_h_ increased from 0.5 to 0.6, and continuously decreased by about 15% when *α*_h_ increased from 0.6 to 0.7. This is due to the increasing sectional depth of SFRELC which provided a restraining effect on the crack growth and strengthened the bearing capacity of SFRELC in the tension zone [24,25]. Meanwhile, the tensile stress of the longitudinal rebars was reduced as presented in relative studies on reinforced SFRC beams [38,39,40].

With the volume fraction of steel fiber *v*_f_ increasing from 0.8% to 1.6%, the elongation of cracks reduced and the crack width became uniform. In the steady development period, the maximum crack width decreased by about 8% when *v*_f_ increased from 0.8% to 1.2% and continuously decreased by about 4% when *v*_f_ increased from 1.2% to 1.6%. This exhibits that the presence of steel fibers in the tension zone of the test beams improved the internal condition of the mid-span section in flexure [35,36,37,38,39,40,49,50].

### 3.2. Failure Pattern

**Figure 5:**
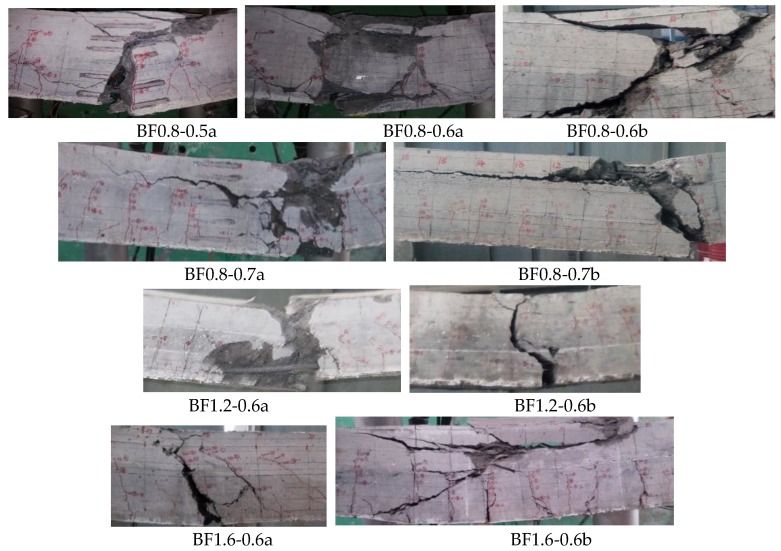
Interesting graphical locations of the fatigue failure patterns of test beams.

As exhibited in Figure 5 showing the interesting graphical locations of the fatigue failure patterns of the test beams, when the fatigue was close to test-beam failure, one of the main cracks grew upward quickly accompanied by the pull-out of steel fibers. In the case of the same *v*_f_ = 0.8%, sudden failure took place on test beam BF0.8-0.5a with *α*_h_ = 0.5 along the main crack with a sudden fracture of the rebar. While in the case of the test beams BF0.8-0.6a/b and BF0.8-0.7a/b with *α*_h_ = 0.6 and 0.7, the conventional concrete crushed in the compression zone almost with the simultaneous fracture of the rebar. This indicates the beneficial effect of the higher SFRELC depth on the improvement of failure brittleness. Meanwhile, the stagger deformation on two sides of the failure section led to a greater splitting force on the horizontal interface between SFRELC and superposed conventional concrete, which resulted in peeling along the horizontal interface on test beams BF0.8-0.7a/b with *α*_h_ = 0.7. 

In case of the same SFRELC depth ratio *α*_h_ = 0.6, the crush of conventional concrete almost with the simultaneous fracture of the rebar occurred on test beams BF0.8-0.6a/b with *v*_f_ = 0.8% and BF1.2-0.6a/b with *v*_f_ = 1.2%, while the crush of conventional concrete only took place on test beams BF1.6-0.6a/b with *v*_f_ = 1.6%. This confirms that the presence of steel fibers in the tension zone of the test beams would not only improve the toughness of failure but also change the failure pattern [16,22]. An obvious phenomenon of more main cracks with shorter extension was observed in BF1.6-0.6b. This is beneficial to the redistribution of tensile force along the span into several main crack sections and the assistance of steel fibers bearing tensile force at the cross-section, and finally contributes to the increase of fatigue lift due to the reduction of tensile stress of the longitudinal tensile rebars. 

Table 3 summarizes the fatigue lift and the characteristics of the failure pattern of the test beams. To get the same maximum crack width under the initial static-load, the actual upper-limit of the fatigue load *P*_max_ was different on the two beams of each group due to the variance of the appearance and growth of cracks. The shorter fatigue life of a test beam existed with a higher *P*_max_, although the same failure pattern appeared in the test beams of a group. This displays that the fatigue life was sensitively affected by the upper-limit of fatigue load.

### 3.3. Compressive Concrete Strain

**Figure 6:**
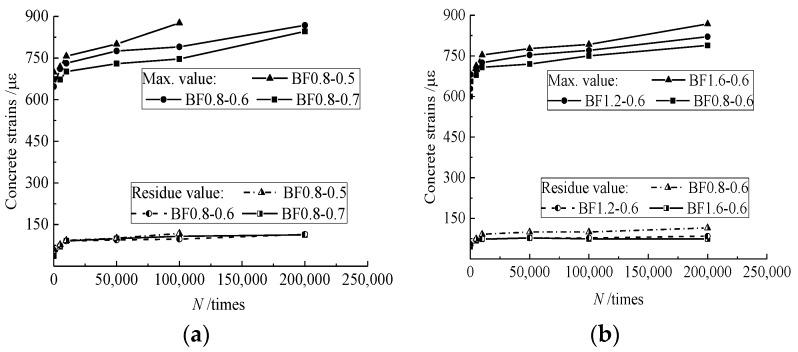
Changes of compressive strain at the top-surface of the mid-span section affected by: (**a**) *α*_h_; and (**b**) *v*_f_.

Under the fatigue load, the total strain of the material increases with the fatigue life in three stages: the first stage is about 5%–10% fatigue life, the strain grows rapidly due to the inherent flaw initiation, such as the presence of air voids and weak regions between aggregates and binder paste; the second stage is about 80% fatigue life, the strain increases slowly and steadily due to the steady development of internal damage; the third stage is about 5%–10% fatigue life, the strain increases fast due to the unstable development of internal damage over a critical limit [37,40,49,50]. In this study, as presented in Figure 6, the increase of concrete strain in the top surface of the compressive zone of the test beams had similar trends as mentioned above in the first two stages with cyclic numbers, while the third stage was not measured due to the defect of measures. During these states, the strain development in the compression zone of the superposed beam did not change with the presence of steel fibers in the tension zone.

In the case of the same *v*_f_ = 0.8%, the initial compressive strain at the top surface of the test beams was approximately equal due to the almost average value of *P*_max_. However, at the same cyclic numbers of fatigue load, the maximum strain gradually reduced with the increase of *α*_h_. This is due to the gradual fracture of steel fibers in the tensile zone with the increase of cyclic numbers of fatigue load. As a result, the neutral axis moved up. The superposed beam with larger *α*_h_ had more steel fibers in the tensile zone subjected to the tensile force to restrain the upward movement of the neutral axis, which gives a relative larger compression zone with a slowly increased strain compared to the beams with smaller *α*_h_. 

In the case of the same *α*_h_ = 0.6, the maximum strain increased with the increase of *v*_f_. This is due to the increased upper-load exerted on the beams. The root is in the higher restraining of the steel fibers on cracks rather than the improvement on the ultimate bearing capacity of the reinforced SFRELC superposed beams [23,24,25,26]. As a result, the steel fibers subjected to higher tensile stress lost the loading function rapidly and the neutral axis moved up quickly, and the compressive strain increased quickly with the reduced compression zone. 

### 3.4. Changes of Mid-Span Deflection

**Figure 7:**
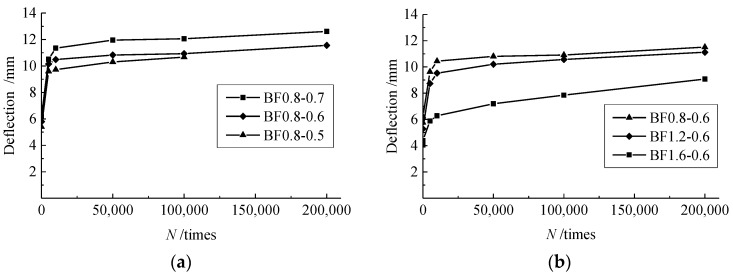
Changes of mid-span deflection affected by: (**a**) *α*_h_; and (**b**) *v*_f_.

The mid-span deflection is a macro index reflecting the sectional deformation of test beams. Similar to the changes in the compressive strain of concrete, as presented in Figure 7, the mid-span deflection of test beams passed through the same stages under the fatigue load. Due to the decrease of the flexural stiffness of the test beams with the increase of *α*_h_ [24,25], the mid-span deflection of the test beams increased with *α*_h_ in the case of the same *v*_f_. Meanwhile, in the case of the same *α*_h_, the confinement of steel fibers to cracks enhanced the integrality of the test beam and benefited the flexural stiffness. This led to a reduction in the mid-span deflection of the test beams with the increasing volume fraction of steel fiber *v*_f_.

## 4. Discussion

The flexural fatigue performance of the reinforced SFRELC superposed beam has the same basic fatigue mechanisms in flexure as the reinforced SFRC beams and the reinforced conventional concrete flexural members [37,38,39,40,49,50]. This should be the compound response of the fatigue compressive strength of conventional concrete, the flexural fatigue behavior of SFRELC, and the fatigue property of the tensile steel bars based on the sectional composition. On the design premise of the tested reinforced SFRELC superposed beams that failed with certain flexural ductility under static loading [23,41], the typical fatigue response with three stages divided into the fast first and rapid third stages accompanied with a second steady stage for the compressive strain of conventional concrete provides the foundation for the flexural fatigue performance of the reinforced SFRELC superposed beams [30,41]. In fact, a similar fatigue response with three stages are presented for the SFRC and steel fiber reinforced lightweight concrete in compression [27,28,29], the SFRC in flexure [35,36], and the steel bars in tension [31]. Therefore, the good properties of materials are the basis for good performances of structural members. On this premise, how to give a full display of the advantages of the materials needs to be given attention, as it is an important issue for the optimization of the composite section within different materials.

For the reinforced SFRELC superposed beams under fatigue loads, the design object should be the utilization of the compression capacity of conventional concrete and the tensile peculiarity of SFRELC to a maximum degree. This has a broader guiding significance for the implication of these kinds of superposed flexural members in civil engineering structures. Meanwhile, the additional phenomena of fatigue failure of the reinforced SFRELC superposed beams may be presented due to the laniation of the horizontal interface between SFRELC and conventional concrete. This can be controlled by the optimization of the SFRELC depth and the volume fraction of steel fibers. If larger shear-tensile stress exists along the horizontal interface due to the higher SFRELC depth, a direct method is to insert vertical reinforcement for the horizontal interface to enhance the entirety of the composite sections [51].

## 5. Prediction of Fatigue Life

### 5.1. Stress Level of Superposed Beam

As the upper-load was determined by the 0.2 mm maximum crack width at the barycenter of the longitudinal rebar, the concrete, steel fiber, and longitudinal rebar were under high stress levels during the fatigue load. The ultimate bending capacity of the reinforced SFRELC superposed beam can be computed with equations as follows [24,41],

(1) $$\alpha_{1}f_{c}bx=f_{y}A_{s}+k_{t}\alpha_{t}\lambda_{f}f_{t}bx_{t},$$

(2) $$M_{u}=f_{y}A_{s}\left(h_{0}-x/2\right)+k_{t}\alpha_{t}\lambda_{f}f_{t}bx_{t}\left(h-x/2-x_{t}/2\right),$$

(3) $$x_{t}=h-x/k_{1}\beta_{1},$$

where, *α*_1_ is the coefficient of the simplified stress figure for compressive concrete, *f*_c_ is the axial compressive strength of conventional concrete, *b* is the sectional width, *x* is the equivalent depth of the compressive zone, *f*_y_ is the yield strength of the longitudinal tensile rebar, *A*_s_ is the sectional area of the longitudinal tensile rebar, *k*_t_ is the adjustive coefficient for the equivalent force of tensile stress, here, *k*_t_ = 1.25; *α*_t_ is the strengthening coefficient of steel fiber to SFRELC, in this study *α*_t_ = 0.31; *λ*_f_ is the steel fiber factor, here, *λ*_f_ = 26.7*v*_f_; *f*_t_ is the tensile strength of concrete with the same grade of SFRELC, *x*_t_ is the equivalent depth of the tensile zone, *k*_1_ is the adjustive coefficient for the equivalent depth of the tensile zone, here *k*_1_ = 0.85; *β*_1_ is the coefficient of the simplified stress figure for compressive concrete, here *β*_1_ = 0.8.

Table 4 presents the computed stress level of fatigue *S* = *P*_max_/*P*_u_. The values are quite high from 0.735 to 0.867. This led to a short fatigue life of test beams due to the unrecoverable plasticity of concrete [49,50].

Normally, the relationship of stress level and fatigue life is represented by the *S*-*N* curve as follows [28],

(4) S=a+blogN,

where *a* and *b* are the coefficients dependent on test data.

**Figure 8:**
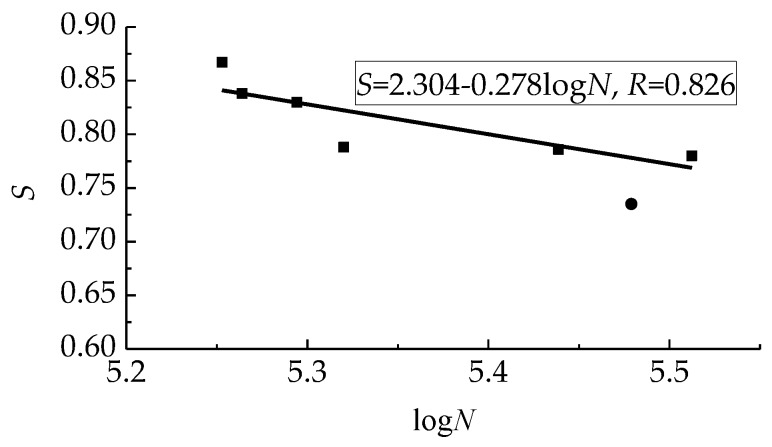
Fitness of *S-N* curve with test data.

Based on test data of this study, *a* = 2.304, *b* = −0.278. The comparison between test data and the computed results is exhibited in Figure 8. A good fit can be given with Pearson’s *R* = 0.826, between the line and test data.

### 5.2. Stress Amplitude of Longitudinal Rebar

According to the China code [41], the stress amplitude of the longitudinal rebar can be computed as follows,

(5) $$\Delta \sigma_{s}^{f}=\sigma_{s,\max}^{f}-\sigma_{s,\min}^{f},$$

(6) $$\sigma_{s,\max}^{f}=\alpha_{E}^{f}M_{\max}^{f}(h_{0}-x_{0})/I_{0},$$

(7) $$\sigma_{s,\min}^{f}=\alpha_{E}^{f}M_{\min}^{f}(h_{0}-x_{0})/I_{0},$$

where, $M_{\max}^{f}$ and $M_{\min}^{f}$ are the maximum and minimum moment under the upper and lower-limit of fatigue loads, $\sigma_{s,\max}^{f}$ and $\sigma_{s,\min}^{f}$ are the maximum and minimum stress of the longitudinal rebar, $\alpha_{E}^{f}$ is the ratio of the modulus of elasticity of the rebar to the modulus of fatigue deformation of conventional concrete, *I*_0_ is the inertia moment of the transformed section, *x*_0_ is the depth of the compression zone of the transformed section, *h*_0_ is the effective depth of the transformed section. 

The sectional characteristic parameters of the transformed section in the condition of unchanged initial centroid can be computed as follows [24],

(8) $$W_{0}=I_{0}/(h-y_{0}),$$

(9) $$y_{0}=\left(\frac{b{\left(h-h_{1}\right)}^{2}}{2}+\alpha_{\mathrm{Ec}}bh_{1}(h-\frac{h_{1}}{2})+(\alpha_{\mathrm{Ec}}-1)A_{s}(h-a_{s})\right)/\left(b(h-h_{1})+\alpha_{\mathrm{Ec}}bh_{1}+(\alpha_{\mathrm{Ec}}-1)A_{s}\right),$$

when $y_{0}\geq h-h_{1}$,

(10) $$I_{0}=\frac{b{\left(h-h_{1}\right)}^{3}}{12}+b(h-h_{1}){\left(y_{0}-\frac{h-h_{1}}{2}\right)}^{2}+\frac{\alpha_{\mathrm{Ec}}b\left({\left(y_{0}-h+h_{1}\right)}^{3}+{\left(h-y_{0}\right)}^{3}\right)}{3}+(\alpha_{\mathrm{Es}}-1)A_{s}{\left(h-a_{s}-y_{0}\right)}^{2},$$

when $y_{0}<h-h_{1}$,

(11) $$I_{0}=\frac{b{\left(h-h_{1}-y_{0}\right)}^{3}}{3}+\frac{by_{0}^{3}}{3}+\alpha_{\mathrm{Ec}}bh_{1}{\left(h-y_{0}-\frac{h_{1}}{2}\right)}^{2}+\frac{\alpha_{\mathrm{Ec}}bh_{1}{}^{3}}{12}+(\alpha_{\mathrm{Es}}-1)A_{s}{\left(h-a_{s}-y_{0}\right)}^{2},$$

where, α_Ec_ is the ratio of the modulus of elasticity between SFRELC and conventional concrete, *y*_0_ is the distance of the centroid to the compressive edge of the transformed section.

The computation results are presented in Table 4. As per China code GB50010 [41], when ρ_f_ = 0.1, $\Delta \sigma_{s}^{f}$ = 162 MPa for the HRB400 rebar. This means that the test stress amplitude was over 38.3%–53.1% of the limit. Therefore, fracture of the longitudinal tensile rebar was commonly inevitable. However, with the assistant of steel fiber in the tension zone, the tensile stress of the rebar should decrease with an increasing volume fraction of the steel fiber. The test beams with *v*_f_ = 1.6% failed without fracture of the longitudinal rebar.

### 5.3. Flexural Stiffness of Normal Section

Flexural stiffness is an important index reflecting the safety of reinforced concrete beams. Based on the principle of minimum flexural stiffness for the computation of the deflection of reinforced flexural members, the flexural stiffness of test beams can be calculated as follows [16,41],

(12) $$B_{s}=0.1065Ml_{0}^{2}/a_{f}$$

where, *M* is the mid-span moment, *l*_0_ is the span, *a*_f_ is the mid-span deflection. 

Based on a previous study, the flexural stiffness *B*_s_ can be computed as follows [16,26],

(13) $$B_{s}=\frac{0.85E_{c}I_{0}}{1+\left(1.16-M_{\mathrm{cr}}/M\right)/\left(6\alpha_{\mathrm{Es}}\rho \right)},$$

(14) $$M_{\mathrm{cr}}=\gamma f_{\mathrm{ft}}W_{0},$$

(15) γ=1.55(0.73+60/h),

where, *E*_c_ is the modulus of elasticity of conventional concrete, *W*_0_ is the elastic resistance moment of the transformed section to tensile edge, *f*_ft_ is the tensile strength of SFRELC, α_Es_ is the ratio of the modulus of elasticity between the rebar and SFRELC, ρ is the reinforcement ratio of longitudinal tensile rebars, *M*_cr_ is the cracking moment of the mid-span section.

Under the upper-load, the tested and calculated values of initial flexural stiffness are presented in Table 5. A good agreement between them is indicated by the average ratio of tested to calculated values of 1.10 with a variation coefficient of 0.056. Meanwhile, the initial flexural stiffness reduced with increasing α_h_, but increased 6.1% and 17.4% in beams with *v*_f_ = 1.2% and 1.6% compared to those with *v*_f_ = 0.8%.

With the increasing cyclic numbers of fatigue load, the compressive strain of conventional concrete and the tensile strains of SFRELC and longitudinal rebar increased. The depth of the compression zone was minimized with the gradual upward extension of cracks. This causes the reduction of the inertia moment of the transformed section and the reduction of the flexural stiffness. Therefore, a revised coefficient *ζ* should be multiplied as follows to compute the flexural stiffness of fatigue $B_{s}^{f}$,

(16) $$B_{s}^{f}=\zeta B_{s},$$

(17) ζ=1−0.04lgN.

**Figure 9:**
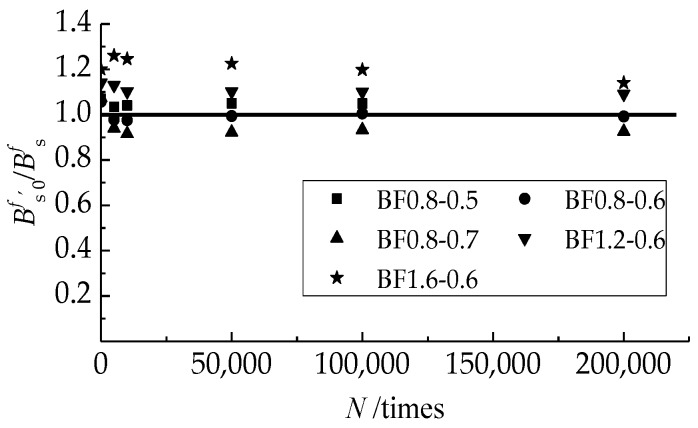
Comparison of tested to computed flexural stiffness at different cyclic numbers of fatigue load.

Table 6 presents the tested and computed values of the flexural stiffness of test beams. The ratios are compared in Figure 9. It gives 1.056 average, with a variation coefficient of 0.099.

**Figure 10:**
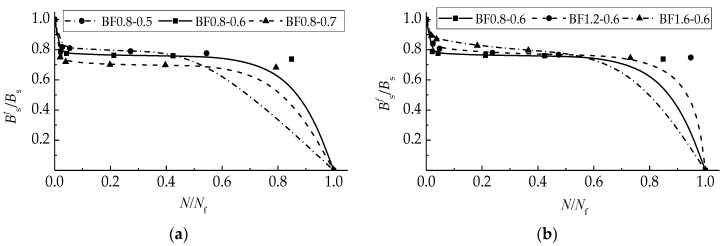
Relative stiffness degradation curve of beam affected by: (**a**) *α*_h_; and (**b**) *v*_f_.

Figure 10 exhibits the trend curves of degenerated flexural stiffness of test beams, where the relative stiffness is the ratio of the stiffness at a cyclic number to the initial stiffness, and the relative life is the ratio *N*/*N*_f_ of a cyclic number to fatigue life.

The curves display three shapes due to the different failure patterns of the test beams. For the test beams in groups BF0.8-0.6, BF0.8-0.7, and BF1.2-0.6, the curves composited by three stages with the first rapidly, the second steady, and the third rapidly. This is similar to that of reinforced concrete flexural members which failed as compressive concrete accompanied by a fracture of the tensile rebars [40,49,50]. For the test beam BF0.8-0.5, the first two stages of the curve are normal, the third drops linearly which represents the sectional fracture without obvious omen. For the test beams in group BF1.6-0.6, the declines in the three stages of the curve are continuous. This exhibits a sustained degeneration of flexural stiffness due to the accumulation of defects in SFRELC and conventional concrete as well as rebars. In this case, the plasticity of the longitudinal tensile rebar has the opportunity to function. Therefore, the controllable failure of the reinforced SFRELC superposed beams can be prospected by the optimal composites of α_h_ and *v*_f_.

## 6. Conclusions

Based on the experimental study, the main conclusion can be drawn as follows:

(1) In the case of the same volume fraction of steel fiber, three failure patterns of test beams under fatigue load took place with the increase of SFRELC depth ratio from 0.5 to 0.7. The first is abrupt along a section of the main crack with the sudden fracture of a tensile rebar, the second is the crush of conventional concrete in the compression zone accompanied with the fracture of the tensile rebar, and the third is only the crush of conventional concrete in the compression zone. It should be noted that the horizontal interface between SFRELC and conventional concrete was laniated during the failure of test beams with an SFRELC depth ratio of 0.7. In this case, the entirety of the composite section should be enhanced.

(2) In the case of the same SFRELC depth ratio, two failure patterns of test beams under fatigue load took place with the increasing volume fraction of steel fiber ranging from 0.8% to 1.6%. Test beams with the steel fiber volume fractions of 0.8% and 1.2% failed with the crush of conventional concrete in the compression zone accompanied with the fracture of the tensile rebar, while the test beams with the steel fiber volume fraction of 1.6% failed with the crush of conventional concrete in the compression zone. This indicated the presence of steel fiber with a larger volume fraction could improve the failure pattern to be more ductile for reinforced SFRELC superposed beams.

(3) The fatigue life was sensitive to the upper limit of the fatigue load. The higher upper limit of the fatigue load led to the higher stress level and larger stress amplitude of the longitudinal rebar. This shortened the fatigue life of the test beams in this study. Formulas are proposed to evaluate the stress level of test beams, the stress amplitude of the longitudinal tensile rebar, and the degenerated flexural stiffness.

(4) The trend curves of fatigue flexural stiffness exhibit the different mechanisms of fatigue failure of test beams. This provides the prospect of controllable failure of reinforced SFRELC superposed beams by the optimal composites of the SFRELC depth ratio and the volume fraction of steel fiber. 

## Figures and Tables

**Table 1:** Basic properties of steel fiber reinforced expanded-shales lightweight concrete (SFRFLC) and conventional concrete.

Concrete	*v*_f_ (%)	Slump (mm)	*f*_fcu_ (MPa)	*f*_fc_ (MPa)	*f*_ft_ (MPa)	*E*_c_ (GPa)
FRFLC	0.8	165	44.9	41.9	3.25	22.8
	1.2	145	43.4	37.1	3.35	22.0
	1.6	140	44.2	41.8	3.73	23.7
Conventional concrete		160	69.1	64.7	2.71	37.0

**Table 2:** Characteristics of test beams.

Beam No.	*b* (mm)	*h* (mm)	*v*_f_ (%)	*α*_h_
BF0.8-0.5a	147	403	0.8	0.5
BF0.8-0.6a	160	403	0.8	0.6
BF0.8-0.6b	156	406	0.8	0.6
BF0.8-0.7a	162	409	0.8	0.7
BF0.8-0.7b	158	406	0.8	0.7
BF1.2-0.6a	153	403	1.2	0.6
BF1.2-0.6b	157	404	1.2	0.6
BF1.6-0.6a	156	402	1.6	0.6
BF1.6-0.6b	154	404	1.6	0.6

**Table 3:** Fatigue life and failure mode of test beams.

Beam No.	*P*_max_ (kN)	*N* (number)	Failure Pattern
BF0.8-0.5a	95	183,654	Abrupt along a section of the main crack with sudden fracture of a rebar.
BF0.8-0.6a	95	196,891	Crush of concrete in the compression zone and fracture of a rebar.
BF0.8-0.6b	90	274,562	
BF0.8-0.7a	90	325,302	Crush of concrete in the compression zone and fracture of a rebar, peeling along the horizontal interface.
BF0.8-0.7b	100	178,997	
BF1.2-0.6a	95	208,963	Crush of concrete in the compression zone and fracture of a rebar.
BF1.2-0.6b	95	213,255	
BF1.6-0.6a	100	245,968	Crush of concrete in the compression zone.
BF1.6-0.6b	95	301,256	

**Table 4:** Stress level of test beams and stress amplitude of longitudinal tensile rebars.

Test Beam	*α*_h_	*P*_max_ (kN)	ρ_f_	*N* (number)	*P*_u_ (kN)	*S*	Average *S*	$\sigma_{s,\max}^{f}\;(\mathrm{MPa})$	$\sigma_{s,\min}^{f}\;(\mathrm{MPa})$	$\Delta \sigma_{s}^{f}\;(\mathrm{MPa})$
BF0.8-0.5a	0.5	95	0.1	183,654	113.3	0.838	0.838	262.6	26.3	236.4
BF0.8-0.6a	0.6	95	0.1	196,891	114.5	0.830	0.808	262.8	26.3	236.5
BF0.8-0.6b	0.6	90	0.1	274,562		0.786		248.9	24.8	224.1
BF0.8-0.7a	0.7	90	0.1	325,302	115.4	0.780	0.823	248.4	24.8	223.5
BF0.8-0.7b	0.7	100	0.1	178,997		0.867		275.9	27.6	248.4
BF1.2-0.6a	0.6	95	0.1	208,963	120.6	0.788	0.788	269.4	26.9	242.4
BF1.2-0.6b	0.6	95	0.1	213,255		0.788		269.4	26.9	242.5
BF1.6-0.6a	0.6	100	0.1	245,968	129.3	0.773	0.754	269.3	26.9	242.3
BF1.6-0.6b	0.6	95	0.1	301,256		0.735		255.8	25.6	230.2

**Table 5:** The measured stiffness of beams under static load and calculated stiffness contrast.

Test Beam	*a*_f_ (mm)	*B*_s_ (×10^12^)		Tested/Calculated
		Tested	Calculated	
BF0.8-0.5a	8.9	3.532	3.302	1.070
BF0.8-0.6a	9.4	3.530	3.274	1.078
BF0.8-0.6b	9.2	3.417	3.301	1.035
BF0.8-0.7a	9.4	3.345	3.276	1.021
BF0.8-0.7b	9.6	3.639	3.225	1.128
BF1.2-0.6a	9.0	3.687	3.233	1.140
BF1.2-0.6b	9.0	3.687	3.233	1.140
BF1.6-0.6a	8.5	4.110	3.382	1.215
BF1.6-0.6b	8.2	4.047	3.412	1.186

**Table 6:** Comparison between measured and calculated values of fatigue stiffness.

Test Beam	Item	Cyclic Numbers of Fatigue Load					
		Initial	5000	10,000	50,000	100,000	200,000
BF0.8-0.5	tested	3.532	2.886	2.861	2.789	2.743	——
	calculated	3.302	2.790	2.750	2.656	2.615	——
BF0.8-0.6	tested	3.474	2.738	2.693	2.649	2.638	2.564
	calculated	3.287	2.800	2.760	2.666	2.625	2.584
BF0.8-0.7	tested	3.492	2.620	2.515	2.447	2.437	2.382
	calculated	3.251	2.788	2.747	2.654	2.613	2.572
BF1.2-0.6	tested	3.687	3.096	2.975	2.874	2.826	2.758
	calculated	3.233	2.740	2.700	2.609	2.569	2.528
BF1.6-0.6	tested	4.078	3.643	3.548	3.372	3.244	3.041
	calculated	3.397	2.890	2.848	2.751	2.708	2.666

## References

[1] Ministry of Housing and Urban-Rural Construction of the People’s Republic of China (2002). Technical Specification of Lightweight Aggregate Concrete.

[2] Standardization Administration of the People’s Republic of China (2010). Lightweight Aggregates and Its Test Methods—Part 1: Lightweight Aggregates.

[3] Zhao M.S., Ma Y.Y., Pan L.Y., Li C.Y. (2015). An Overview of Study on Natural Lightweight Aggregate for Concrete in China.

[4] Ma Y.Y., Fan Y.J., Pan L.Y., Li C.Y. (2015). Study and Application of Cinder as Lightweight Aggregate of Concrete in China.

[5] Zhao M.S., Zhang X.Y., Song W.H., Li C.Y., Zhao S.B. (2018). Development of steel fiber reinforced expanded-shale lightweight concrete with high freeze-thaw resistance. Adv. Mater. Sci. Eng..

[6] Ministry of Housing and Urban-Rural Construction of the People’s Republic of China (2006). Technical Specification for Lightweight Aggregate Concrete Structures.

[7] Hassanpour M., Shafigh P., Mahmud H.B. (2012). Lightweight aggregate concrete fiber reinforcement—A review. Constr. Build. Mater..

[8] Zhao S.B., Li C.Y., Qian X.J. (2011). Experimental study on mechanical properties of steel fiber reinforced full lightweight concrete. Geotech. Special Pub..

[9] Pan L.Y., Yuan H., Zhao S.B. (2011). Experimental study on mechanical properties of hybrid fiber reinforced full lightweight aggregate concrete. Adv. Mater. Res..

[10] Li C.Y., Chen H., Zhao S.B. (2012). Mechanical properties of steel fiber reinforced light-aggregate concrete. Adv. Mater. Res..

[11] Zhao M.L., Zhao M.S., Chen M.H., Li J., Law D. (2018). An experimental study on strength and toughness of steel fiber reinforced expanded-shale lightweight concrete. Constr. Build. Mater..

[12] Zhao S.B., Zhao M.S., Zhang X.Y., Peng Z.J., Huang T.H. (2019). Study on complete stress-strain curves of steel fiber reinforced lightweight-aggregate concrete under uniaxial compression. J. Build. Struct..

[13] Zhao M.S., Zhang B.X., Shang P.R., Fu Y., Zhang X.Y., Zhao S.B. (2019). Complete stress-strain curve of self-compacting steel fiber-reinforced expanded-shale lightweight concrete under uniaxial compression. Materials.

[14] Zhao S.B., Li C.Y., Zhao M.S., Zhang X.Y. (2016). Experimental study on autogenous and drying shrinkage of steel fiber reinforced lightweight-aggregate concrete. Adv. Mater. Sci. Eng..

[15] Zhao M.S., Zhang X.Y., Yan K., Fei T., Zhao S.B. (2018). Bond performance of deformed rebar in steel fiber reinforced lightweight-aggregate concrete affected by multi-factors. Civ. Eng. J..

[16] Zhao M.S., Su J.Z., Shang P.R., Zhao S.B. (2019). Experimental study and theoretical prediction of flexural behaviors of reinforced SFRELC beams. Constr. Build. Mater..

[17] Li X.K., Li C.Y., Zhao M.L., Yang H., Zhou S.Y. (2019). Testing and prediction of shear performance for steel fiber reinforced expanded-shale lightweight concrete beams without web reinforcements. Materials.

[18] Li C.Y., Yang H., Liu Y., Gao K. (2013). Flexural behavior of reinforced concrete beams superposing with partial steel fiber reinforced full-lightweight concrete. Appl. Mechan. Mater..

[19] Pei S.W., Du Z.H., Li C.Y., Shi F.J. (2013). Research of punching performance of reinforced SFRLAC superposed two-way slabs. Appl. Mechan. Mater..

[20] Nes L.G., Øverli J.A. (2015). Structural behaviour of layered beams with fibre-reinforced LWAC and normal density concrete. Mater. Struct..

[21] Iskhakov I., Ribakov Y. (2007). A design method for two-layer beams consisting of normal and fibered high strength concrete. Mater. Des..

[22] Holschemacher K., Iskhakov I., Ribakov Y., Mueller T. (2012). Laboratory tests of two-layer beams consisting of normal and fibered high strength concrete: Ductility and technological aspects. Mechan. Adv. Mater. Struct..

[23] Li C.Y., Zhao S.B., Chen H., Gao D.Y. (2015). Experimental study on flexural capacity of reinforced SFRFLC superposed beams. J. Build. Struct..

[24] Li C.Y., Ding X.X., Zhao S.B., Zhang X.Y., Li X.K. (2016). Cracking resistance of reinforced SFRFLC superposed beams with partial ordinary concrete in compression zone. Open Civ. Eng. J..

[25] Li C.Y., Zhao Z.F., Ding X.X., Zhao S.B. (2016). Experimental study on crack width of reinforced HSC-on-SFRFLC superposed beams. Adv. Eng. Res..

[26] Pan L.Y., Shang Y.Q., Kang X.X., Li C.Y. (2015). Experimental research on flexural stiffness of reinforced steel fiber reinforced full-lightweight concrete superposed beams. J. North China Univ. Water Resour. Electric Power (Nat. Sci. Ed.).

[27] Goel S., Singh S.P. (2014). Fatigue performance of plain and steel fibre reinforced self compacting concrete using S-N relationship. Eng. Struct..

[28] Zhang C., Gao D.Y., Gu Z.Q. (2017). Fatigue behavior of steel fiber reinforced high-strength concrete under different stress levels. IOP Conf. Series: Mater. Sci. Eng..

[29] Choi S.J., Mun J.S., Yang K.H., Kim S.J. (2016). Compressive fatigue performance of fiber-reinforced lightweight concrete with high-volume supplementary cementitious materials. Cem. Concr. Compos..

[30] Lantsoght E.O.L., van der Veen C., de Boer A. (2016). Proposal for the fatigue strength of concrete under cycles of compression. Constr. Build. Mater..

[31] Hawileh R., Rahman A., Tabatabai H. (2010). Evaluation of the low-cycle fatigue life in ASTM A706 and A615 grade 60 steel reinforcing bars. J. Mater. Civ. Eng..

[32] De Corte W., Boel V., Helincks P., De Schutter G. (2016). Fatigue assessment of a lightweight steel-concrete bridge deck concept. Bridge Struct..

[33] Chen H.J., Liu T.H., Tang C.W., Tsai W.P. (2011). Influence of high-cycle fatigue on the tension stiffening behavior of flexural reinforced lightweight aggregate concrete beams. Struct. Eng. Mechan..

[34] Li C.Y., Nie X., Kang X.X., Peng C., Zhang X.Y., Zhao S.B. (2017). Experimental study on shear fatigue behavior of reinforced SFRFLC superposed beams. J. North China Univ. Water Resour. Electric Power.

[35] Singh S.P., Kaushik S.K. (2003). Fatigue strength of steel fibrous concrete in flexure. Cem. Concr. Compos..

[36] Germano F., Tiberti G., Plizzari G. (2016). Post-peak fatigue performance of steel fiber reinforced concrete under flexure. Mater. Struct..

[37] Batson G. (1972). Flexural fatigue strength of steel fiber reinforced concrete beams. ACI J..

[38] Goel S., Singh S.P., Singh P. (2012). Felxural fatigue strength and failure probability of self compacting fibre reinforced concrete beams. Eng. Struct..

[39] Banjara N.K., Ramanjaneyulu K. (2018). Experimental investigations and numerical simulations on the flexural fatigue behaviors of plain and fiber-reinforced concrete beams. J. Mater. Civ. Eng..

[40] Parvez A., Foster S.J. (2015). Fatigue behavior of steel-fiber-reinforced concrete beams. J. Struct. Eng..

[41] Ministry of Housing and Urban-Rural Construction of the People’s Republic of China (2011). Design Code for Concrete Structures.

[42] General Administration of Quality Supervision, Inspection and Quarantine of the People’s Republic of China (2007). Common Portland Cement.

[43] General Administration of Quality Supervision, Inspection and Quarantine of the People’s Republic of China (2005). Fly Ash Used for Cement and Concrete.

[44] Ministry of Housing and Urban-Rural Construction of the People’s Republic of China (2011). Specification for Mix Proportion Design of Ordinary Concrete.

[45] Ministry of Housing and Urban-Rural Construction of the People’s Republic of China (2015). Steel Fiber Reinforced Concrete.

[46] Ministry of Housing and Urban-Rural Construction of the People’s Republic of China (2002). Standard for Test Method of Performance on Ordinary Fresh Concrete.

[47] Ministry of Housing and Urban-Rural Construction of the People’s Republic of China (2002). Standard for Test Method of Mechanical Properties on Ordinary Concrete.

[48] Ministry of Housing and Urban-Rural Construction of the People’s Republic of China (2012). Standard for Testing Method of Concrete Structures.

[49] Song Y.P., Zhao S.B., Wang R.M., Li S.Y. (1994). Full-range nonlinear analysis of fatigue behaviors of reinforced concrete structures by finite element method. Acta Oceanol. Sin..

[50] Zhao S.B. (1999). Experimental study on fatigue behaviors of reinforced concrete plates. J. Basic Sci Eng..

[51] Zhao S.B. (1998). Experimental research on shear behavior of reinforced concrete composite beams under uniformly distributed load. China Ocean Eng..

